# Hemisection versus conventional extraction as interceptive treatment in congenitally missing mandibular second premolars: a randomised controlled split-mouth trial

**DOI:** 10.1093/ejo/cjaf043

**Published:** 2025-06-12

**Authors:** Sarah Abdul Jabbar, Shaker Nawaia, Vini Rughwani, Ken Hansen, Julia Naoumova

**Affiliations:** University Clinic of Orthodontics, Gothenburg, Public Dental Service, Region Västra Götaland, 54130, Sweden; Specialist clinic of Orthodontics, Skövde Public Dental Service, Region Västra Götaland, 41346, Sweden; Specialist clinic of Orthodontics, Skövde Public Dental Service, Region Västra Götaland, 41346, Sweden; University Clinic of Orthodontics, Gothenburg, Public Dental Service, Region Västra Götaland, 54130, Sweden; University Clinic of Orthodontics, Gothenburg, Public Dental Service, Region Västra Götaland, 54130, Sweden; Department of Orthodontics, Institute of Odontology, Sahlgrenska Academy, University of Gothenburg, 40530, Sweden

**Keywords:** hypodontia, tooth agenesis, aplasia, primary molar

## Abstract

**Background:**

The congenital absence of mandibular second premolars is a common anomaly requiring careful treatment planning. Conventional extraction of the primary molar often causes spontaneous space closure but may lead to mesial tipping of adjacent teeth. Hemisection offers an alternative to control tooth movement and reduce tipping. However, evidence comparing hemisection and conventional extraction, particularly on space closure and tooth angulation, is limited.

**Objectives:**

To compare conventional extraction with hemisection of the mandibular primary second molars in terms of space closure, tooth angulation, complications and associated economic implications in patients with congenital absence of mandibular second premolars.

**Trial design:**

prospective, randomised longitudinal split-mouth.

**Materials and Methods:**

Patients with bilateral agenesis of the second mandibular molars and unerupted second molars were included and randomly allocated to either extraction or hemisection on the left or right side of the mandible. Clinical and radiographic examinations were conducted at baseline (T1) and after a mean follow-up period of 4.2 years (T2). Measurements of the residual spaces and tooth angulation of the mandibular first molar and premolar following extraction were blinded assessed on panoramic radiographs and cast models. The number of visits, chair time, social costs, and direct and indirect costs were calculated using cost minimisation analysis.

**Results:**

A total of 40 patients (25 boys and 15 girls) with a mean age of 10.03 ± 1.07 years at T1 participated. No patient was lost to follow-up. The residual space between the first permanent molar and the first permanent premolar was 2.04 ± 1.67 mm for hemisection and 2.39 ± 1.86 mm for extraction (p = 0.053). A larger residual space was observed between the first permanent premolar and the canine on the hemisection side (1.80 ± 1.01 mm) than on the extraction side (1.55 ± 0.92 mm), (p = 0.045). No difference was found between the interventions regarding the angulation of the first permanent molar (p = 0.0914) or the angulation of the first permanent premolar (p = 0.7812). Hemisection resulted in significantly more complications (p = 0.0176) and was associated with substantially higher material costs, more chair time and higher indirect costs than conventional extraction (p < 0.0001).

**Conclusion:**

Hemisection is not recommended as an interceptive extraction option for patients with congenitally missing mandibular second premolars, as only minimal, clinically irrelevant differences were observed compared with conventional extraction. Moreover, hemisection is associated with increased costs and a higher risk of complications.

**Trial Registration:**

The trial was registered with https://www.researchweb.org/is/sverige, registration number: 967125.

## Introduction

Congenital absence of one or more permanent teeth, known as agenesis or hypodontia is a common congenital anomaly in the human dentition. The prevalence of agenesis in the Scandinavian population ranges between 6.1%–8.2%, with mandibular second premolars the most frequently missing teeth in the permanent dentition, excluding third molars [1–4]. Agenesis of the mandibular second premolar should not be diagnosed before the age of nine to minimise false positive diagnoses, as there are tooth buds with late onset of mineralisation [1, 5]. When deciding to treat in cases of hypodontia, factors such as the age of the patient, the type of occlusion and the status of the primary tooth need to be considered. One treatment option is to extract the deciduous molar and await spontaneous space closure or active space closure with orthodontic appliances.

Early interceptive extraction of the deciduous second molar has the advantage of promoting spontaneous space closure during the eruption of the second molar. This mesial migration of the first permanent molar has been shown to close 84% of the space [6]. The space closure occurs via parallel movement and tipping of the adjacent teeth [6, 7]. The extent of the tipping depends on the time of the extraction and on the vertical growth, demonstrating the importance of performing the interceptive extraction at the right time [8]. An alternative to conventional extraction is hemisection or slicing of the deciduous second molar and extracting the distal and mesial segments on two different occasions. In a study by Northway (2004), hemisection of the primary second molar was used as the first step in space closure, followed by the insertion of fixed appliances. Valencia et al. (2004) demonstrated that a controlled slicing technique, which encourages bodily mesial drift of the permanent first molar, achieved approximately 80% space closure within one year without significant mesial rotation or midline shift. This approach showed a success rate of about 90% when applied at an early age (8–9 years), whereas extraction alone resulted in average to poor outcomes in 75% of cases [8, 9].

Even though hemisection seems more advantageous in theory and according to the few studies in the literature that have assessed the method, there are to date no randomised controlled trials that compare conventional extraction and hemisection. According to a systematic review, there is a knowledge gap as there are no prospective longitudinal studies comparing conventional extraction with hemisection of the second deciduous molar in patients with agenesis of the second mandibular premolar [10]. Furthermore, reports of symptoms and complications following hemisection and conventional extraction are missing.

In Sweden, children and adolescents receive free dental care up to the age of 20 . However, the healthcare sector faces several constraints, including limited staffing, time, facilities, equipment, and expertise. Failing to adhere to cost effectiveness principles can lead to unsustainable financial burdens. Therefore, health economic evaluations play a critical role in addressing these challenges by systematically assessing the costs and outcomes of healthcare interventions to determine their overall value and to guide resource allocation. Four primary methods are widely recognised and utilised in economic evaluations [11]: cost-effectiveness, cost-minimisation, cost-utility and cost-benefit analysis. When allocating resources to dental care and orthodontics, both the clinical effectiveness of treatment procedures and their relative costs should be considered [12]. The societal costs of hemisection and conventional tooth extraction have not previously been compared.

This study, designed using the PICO framework, aims to evaluate patients with congenital absence of mandibular second premolars (Population). It compares hemisection of the second primary mandibular molar (Intervention) to conventional extraction (Comparison). The primary outcome is space closure, while secondary outcomes include angulation of adjacent teeth, complications, and cost-effectiveness (Outcome), aiming to determine the most favourable treatment approach.

The null hypothesis was that there are no differences in space closure, angulation of the adjacent teeth, complications or in costs between conventional extraction or hemisection.

## Materials and methods

### Trial design

This was a single-centre, single blinded, split-mouth randomised controlled trial (RCT). The study was carried out in accordance with the Declaration of Helsinki and ethical approval was granted from the Regional Ethical Review Board at the University of Gothenburg (reg. no.558-17). Written informed consent and assent was obtained from all participants and their parents/guardians. This trial was registered prior to commencement, ensuring transparency and adherence to ethical guidelines. No changes were made to the trial protocol after registration or study initiation. The study was conducted in accordance with CONSORT guidelines to ensure methodological rigor and reliability of the findings.

### Participants, setting, and eligibility criteria

All consecutive patients who fulfilling the eligibility criteria from nine public dental clinics in the Skaraborg region in central Sweden between 2017 and 2020 were referred to the orthodontic clinic in Skövde, Sweden.

The inclusion criteria were:

- Age 9-12 years.
- Bilateral agenesis of the mandibular second premolars.
- Bilateral persisting deciduous second mandibular molars.
- Bilateral presence and unerupted mandibular second molars.

The exclusion criteria were:

- Angle class II:2 (as mandibular extractions could potentially deepen the bite)
- Generalised spacing in the mandible (defined as ≥ 8mm of spacing measured from the mesial right primary second molar to the mesial left primary second molar).
- Cleft lip and palate or other craniofacial deformities.

### Sample size

The sample size was calculated based on an alpha significance level of 0.05 and a beta of 0.1 to achieve 90% power to detect a clinically meaningful difference of 2 mm (± 1 mm) in residual space between the two extraction modalities. The power analysis showed that 30 patients were required. To account for a possible dropout rate of 30%, the sample size was increased to a total of 40 patients.

### Randomisation

Stratified randomisation was applied to assign the extraction method for each patient, including the specific tooth to be extracted or hemisectioned and the sequence in which the procedures would occur. The randomisation sequence list was prepared using an online platform (https://www.graphpad.com/quickcalcs/randomize1.cfm by one of the authors (JN) who was not involved in the enrolment or treatment of the patients. Allocation concealment was ensured by using sequentially numbered, opaque, sealed envelopes, which were prepared in advance of the trial by the same author responsible for treatment and order assignment.

### Interventions

The extractions were performed before the eruption of the permanent second mandibular molars, as this timing has been previously shown to result in mesial drift of the first and second permanent molars [6].

#### Hemisection

**Figure. 1.**
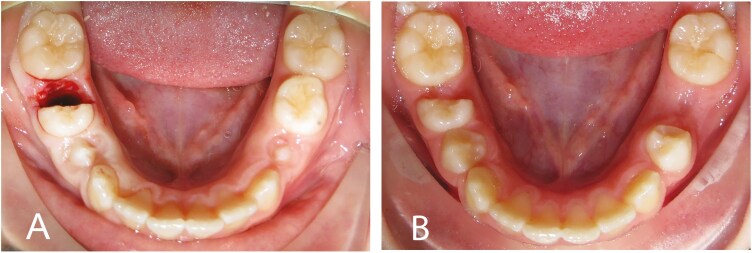
**A**, Hemisection and extraction of the distal root of right second primary molar. **B**, 6 months later before extraction of the mesial root, 2 mm of space was measured between the distal surface of the first permanent molar and the mesial surface of the divided deciduous molar before extraction of the mesial root. The left second primary molar was conventionally extracted.

Local anaesthesia was applied to the extraction area prior to the treatment. The deciduous tooth was divided with a Zekrya surgical bur in the buccal-lingual direction through the bifurcation of the tooth. The distal part of the tooth (including root and crown) was extracted with extraction forceps. The remaining mesial part of the tooth was left without surgical dressing or endodontic treatment (Fig. 1A). Hemostasia was ensured using a surgical compress and applying pressure to the extraction area. Postoperative information was given to the patients including the recommendation to take two painkillers if needed. The patients were given appointments every eight weeks to measure the residual space between the most mesial point on the first permanent molar and the distal surface of the hemisected deciduous molar. The space was measured intraorally with a digital calliper by the same operator at every check-up. When the space was less than 2 mm, the mesial portion of the deciduous tooth was extracted (Fig. 1B).

#### Conventional extraction

Local anaesthesia was applied to the extraction area prior to treatment. The deciduous tooth was extracted with extraction forceps (Fig. 1B). Hemostasia was ensured by using a surgical compress and applying pressure to the extraction area. Postoperative information was given to the patients including the recommendation to take two painkillers if needed.

The hemisection and the conventional extraction were performed on the same patient 10-14 days apart when the patients had no postoperative symptoms from the previous intervention.

At baseline (T1), a clinical examination was performed, including extraoral and intraoral photographs and a panoramic radiograph. The residual space was assessed at the 4-year follow-up (T2), by which time the second molars were anticipated to be fully erupted. At T2, a clinical examination was conducted, along with extraoral and intraoral photographs, a panoramic radiograph, and study casts.

### Primary outcome measure

#### Residual space

Residual space was assessed on the cast models using four outcome measures: molar premolar distance (MPD), premolar canine distance (PCD), and canine-incisor distance (CID) (Table 1). Measurements were made using a digital calliper with 0.1 mm accuracy (Karl Hammacher GmbH, REF HSL 246-15). To measure spaces smaller than 1.00 mm, a manual interproximal measuring instrument was used with thicknesses of 0.10 mm, 0.15 mm, 0.20 mm, 0.25 mm, 0.30mm, 0.40 mm, 0.50 mm and 1.0 mm (Intensiv IPR DistanceControl, REF. IPR-DC set).

**Table 1.** Cast model outcome measurements and definitions.

Measurement	Definition
Crossbite	The buccal cusp of the maxillary tooth occludes lingually to the buccal cusp of the corresponding mandibular tooth.
Scissors bite	The lingual cusp of the maxillary tooth occludes buccally to the buccal cusp of the mandibular corresponding tooth.
Occlusion in the premolar area	The first and/or second premolar in the upper jaw has antagonist contact.
Occlusion of the second molar in the upper jaw	The second permanent molar in the upper jaw has antagonist contact.
Interproximal space (Molar-premolar distance, MPD)	The distance in millimetre between the most prominent mesial surface of first permanent molar and the most prominent distal surface of the first permanent premolar.
Interproximal space (Premolar-canine distance, PCD)	The distance in millimetre between the most prominent mesial surface of the first permanent premolar and the most prominent distal surface of the permanent canine.
Interproximal space (Canine-incisor distance, CID)	The distance in millimiter between the most prominent mesial surface of the permanent canine and the most prominent distal surface of the permanent lateral incisor.

Recordings assessed on the cast models were: crossbite, scissor bite, occlusion in the premolar and second molar area (Table 1).

### Secondary outcome measures

#### Angulation

**Figure 2.**
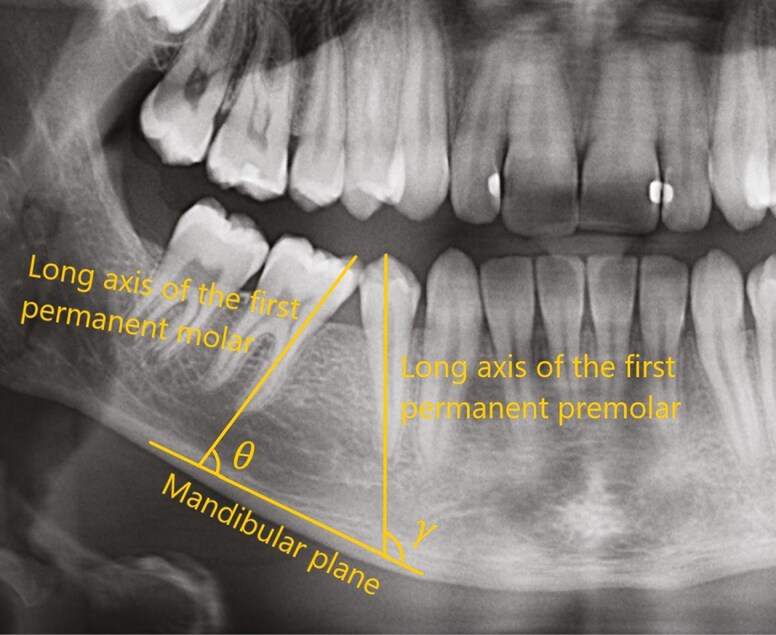
Mandibular plane: reference line. Molar and premolar angle (θ, γ): The angle between the long axis of the teeth and the mandibular plane.

**Figure 3.**
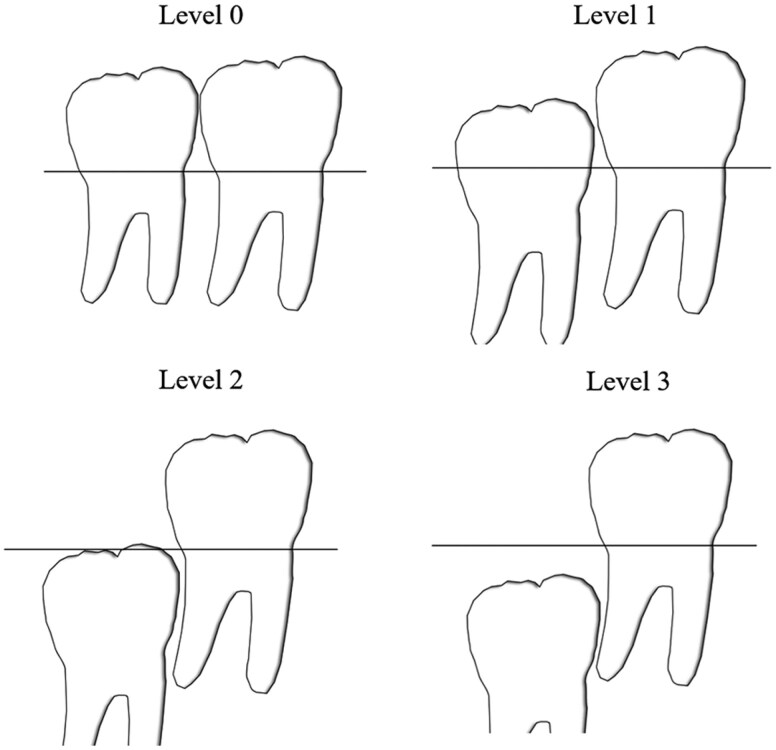
Assessment of the vertical relationship between the first permanent molar and the second permanent molar divided into the four illustrated levels. Level 0: Second permanent molar fully erupted. Level 1: The crown of the second permanent molar is level with the crown of the first permanent molar. Level 2: Crown of the second permanent molar between the cementoenamel junction and furcation of the first permanent molar. Level 3: Crown of the second permanent molar between the furcation and apex of the first permanent molar. Adapted from the study by Aldahool et al. [15].

The angulation of the mandibular first molar and premolar was evaluated using two outcome measures: molar angle and premolar angle (Table 2, Fig. 2). These angles were measured on the panoramic radiographs taken at T2 using the tracing program FACAD® (Ilexis AB, Linköping, Sweden). Recordings on the panoramic radiographs taken at T1 were vertical relationship and root development stage of second permanent molar and first permanent premolar and presence of mandibular third molars (Table 2, Fig. 3).

**Table 2.** Radiographic outcome measurements and definitions

Measurement	Definition
Molar angle (θ)	The angle between the long axis of the permanent molar and the mandibular plane, θ. (Figure 2).
Premolar angle (γ)	The angle between the long axis of the permanent premolar and the mandibular plane, γ. (Figure 2).
Root development stage	The root development stage of the second permanent molar and the first permanent premolar (T0) according to Nollas’ stages of permanent teeth development [13].
Vertical relation	The vertical relation between the second permanent molar and the first permanent molar (T0) was divided into four different levels according to a method used by Aldahool et. al [14],. (Figure 3).
Third molar	Presence of the third molar in the lower jaw.

The panoramic radiographs were taken using two different X-ray machines: Planmeca ProMax 2D® (Planmeca Inc., Helsinki, Finland), with the exposure settings of 62 kV and 5mA, and Orthopantomograph OP200 D® (Instrumentarium Oy, Tuusula, Finland), with the exposure settings of 66 kV and 5mA. The patients were positioned according to the standard positioning technique [13]. Each panoramic radiograph was calibrated using tooth size measurements obtained from the patient’s cast model.

#### Symptoms and complications

Data on complications during the extractions and post-extraction symptoms were collected from the patient’s dental records. Symptoms such as pain, swelling, bleeding, and infection, along with complications like root fracture and ankylosis, were recorded. For the extraction side, secondary outcome measures were recorded up to one month after extraction, while on the hemisection side, they were recorded one month after the extraction of the mesial root.

### Blinding

This study was single-blinded, with two operators performing the clinical interventions. All measurements were conducted by an independent examiner who was blinded to the intervention administered to the patients.

#### Economic evaluation

A cost minimisation analysis was performed under the assumption that the treatment options resulted in equivalent clinical outcomes. Direct costs included expenditure directly associated with the clinical procedures, including material costs, facility and equipment usage, maintenance, cleaning, and staff salaries. These costs were derived from the monthly financial records maintained by the specialist orthodontic clinic in Skövde, Sweden. Indirect costs were defined as income loss incurred by parents due to absence from work to accompany their children to appointments. This included time spent at the clinic as well as travel time to and from the facility. Data on the average Swedish income estimated at €17.14 per hour, were sourced from Statistics Sweden (www.scb.se). For the analysis, the average travel time per visit to the clinic was assumed to be 60 minutes. The total treatment time was calculated as the sum of all appointment durations for each treatment group, while the total duration of parents’ absence from work was determined by combining the total treatment time with the travel time for all visits. Societal costs were calculated by summing up the direct and indirect costs and dividing the sum with the number of participants in each group. All costs were expressed in Euros (€) based on 2025 values, using a conversion rate of SEK 100 = €8.7, derived from the average exchange rate (www.xe.com).

### Statistical analysis

Statistical analyses were conducted using SAS (version 9.3). Means and standard deviations (SD) were calculated for numerical variables. The normality of the data distribution was assessed by the Shapiro–Wilk test, which confirmed that the data followed a normal distribution, allowing for the use of parametric tests. Differences in means between the sides and time points were tested by paired t-tests while gender differences were tested with unpaired t-tests. Multiple regression analysis was used to detect variables associated with the size of the residual space. One-way analysis of variance (ANOVA) with the Student-Newman-Keuls post hoc test was used for variables with three or more categories.

Differences in mean costs between hemisection and conventional extraction were assessed using the comparison of means tool available in MedCalc Software (https://www.medcalc.org/calc/comparison_of_means.php).

The significance level was set at P < 0.05.

## Error of method

To assess measurement precision and reliability, the same examiner remeasured 40 randomly chosen panoramic radiographs and 20 randomly chosen cast models. Dahlberg’s formula was used to calculate the mean errors for the linear and angular measurements. The linear measurements ranged from 0.04 to 0.06 mm on the cast models and between 0.15 to 0.24 mm on the panoramic radiographs. The angular measurements on the panoramic radiographs ranged from 0.86 to 1.01°. No significant differences were observed between the first and the second measurement.

## Results

**Figure 4.**
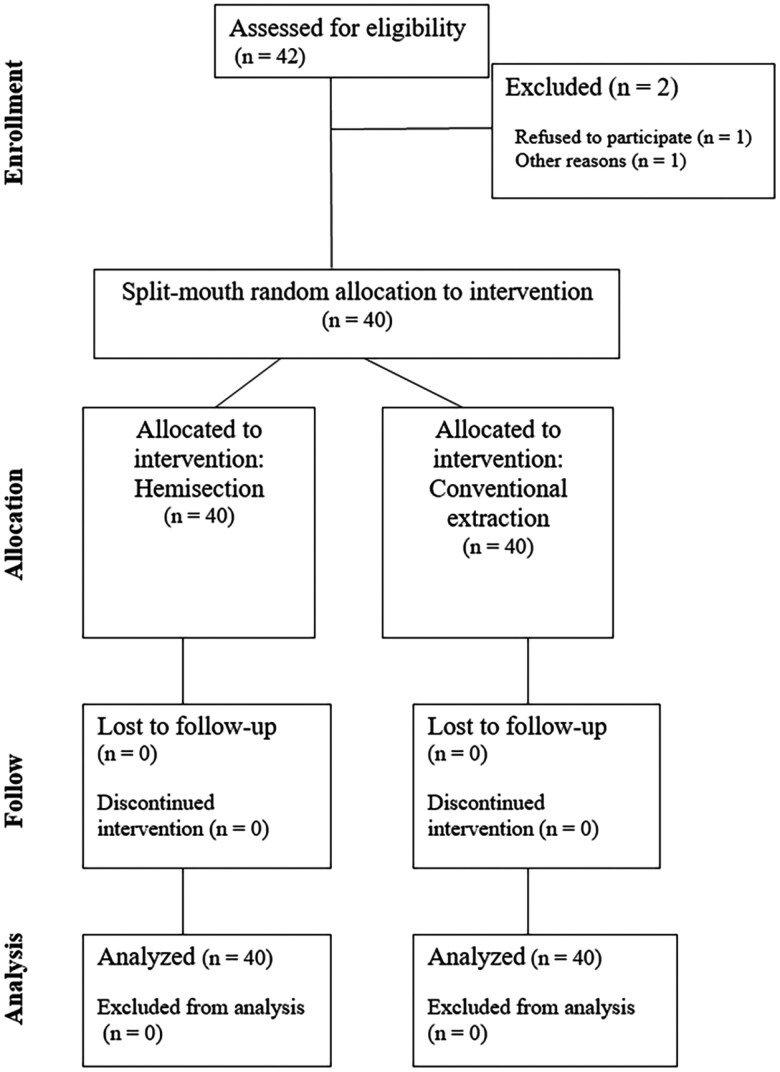
Flowchart describing the protocol and number of patients included (n). No patients were lost to follow-up.

Forty patients: 25 boys (mean age ± SD: 10.56 ± 1.68) and 15 girls (mean age ± SD: 10.06 ± 0.73) with bilateral congenitally missing mandibular permanent second premolar participated in the study. No patients were lost during the follow-up period (Fig. 4). The mean ± SD follow-up time was 4.2 ± 0.6 years. For the hemisection side, the mean time between the extraction of the distal segment and the mesial segment was 10.58 ± 4.71 months.

At T1, there were no differences in root development of either the second molar (p = 0.2532) or the first premolar (p = 0.3729) or in the vertical position of the second molar (p = 1.0000) (Fig. 3) between the hemisection side and the extraction side (Table 3). At T2, crossbite was observed in 2.5% (n = 1) and scissor bite in 2.5% (n = 1) of the patients on the extraction side, while on the hemisection side, 5% (n = 2) had crossbite and 5% (n = 2) scissor bite. Cross-bite and scissor-bite involved either maxillary first or second premolar and mandibular first molar or first premolar.

**Table 3.** The baseline distribution of root developmental stages according to Nolla for the second molar and first premolar [14] and the vertical position of the second molar, presented as the number of teeth (N) and percentage (%).

Variable	Hemisection (N = 40) N, (%)	Extraction (N = 40) N, (%)	P-value
**Second molar root development**			
4	-	1, (2.5%)	0.2532
5	1, (2.5%)	2, (5%)	
6	9, (22,5%)	9, (22.5%)	
7	23, (57.5%)	23, (57.5%)	
8	7, (17.5%)	5, (12.5%)	
**First premolar root development**			
6	4, (10%)	4, (10%)	0.3729
7	22, (55%)	22, (55%)	
8	12, (30%)	13, (32.5%)	
9	2, (5%)	1, (2.5%)	
**Second molar vertical position**			
0	-	1, (2.5%)	1.0000
1	4, (10%)	2, (5%)	
2	29, (72%)	30, (75%)	
3	7, (17.5%)	7, (17.5%)	

All first mandibular molars, except one, were in occlusal contact with the maxillary first molar on the extraction and hemisection side (100% and 97.5% of the teeth, respectively). A similar situation was seen for the first premolar on the extraction and the hemisection side (90% and 82.5% of the teeth, respectively).

### Primary outcome

#### Residual space

**Figure 5.**
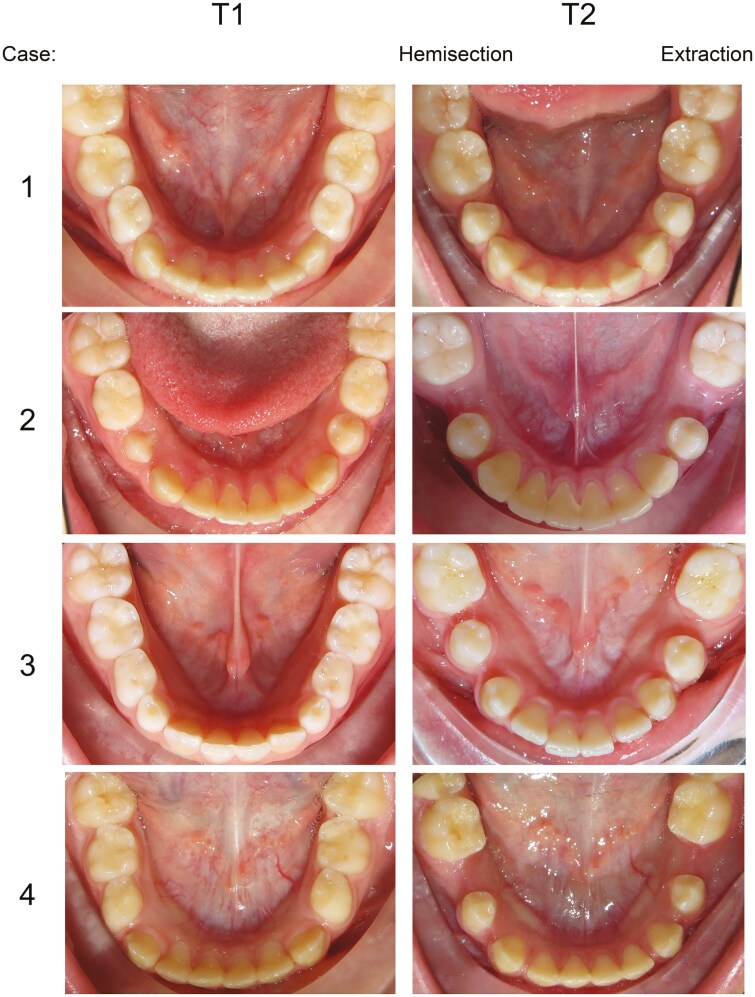

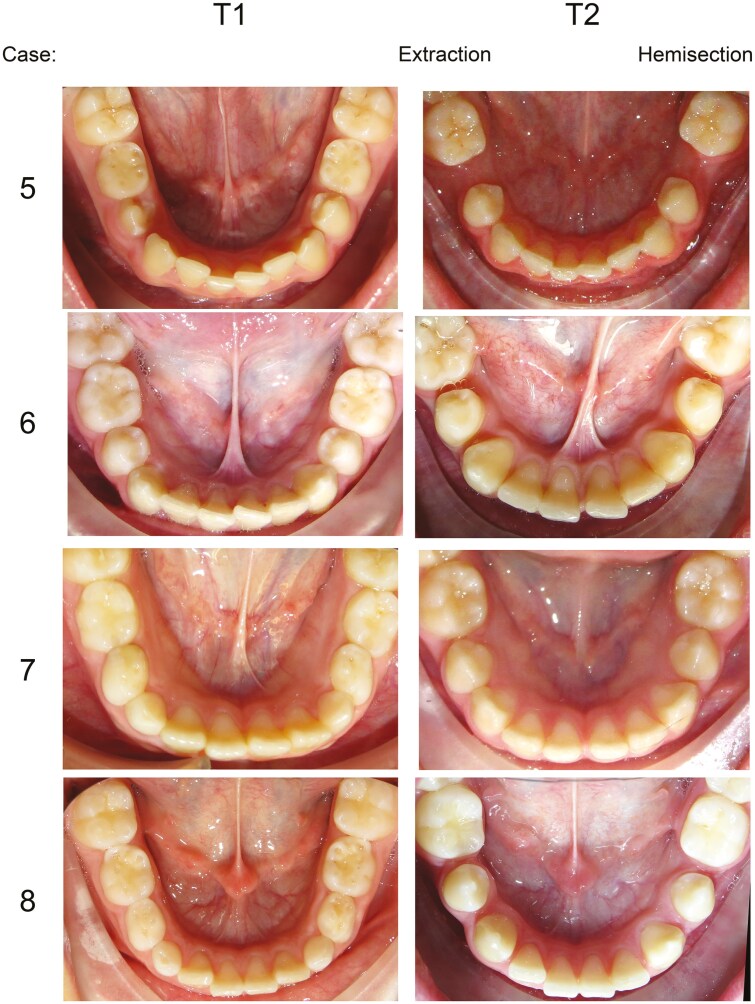
Eight patients at baseline (T1) and at the 4.2-year follow-up (T2). In the cases 1-4, hemisection was performed on the right side and extraction on the left side. In the cases 5-8, extraction was made on the right side and hemisection on the left side.

The mean residual space (Table 4) between the first permanent molar and premolar (MPD) was statistically significantly smaller at the hemisection side than the conventional extraction side (2.04 ± 1.67 mm and 2.39 ± 1.86 mm, respectively, p = 0.0525). However, the mean residual space between the first permanent premolar and the canine (PCD) was statistically significantly larger at the hemisection side than the conventional extraction side (1.80 ± 1.01mm and 1.55 ± 0.92mm, respectively, p = 0.0455). Thus, when the total residual spaces (molar to canine) were compared, no significant difference was seen between the hemisection and extraction sides (p = 0.6446) (Table 4 and Fig. 5). No gender differences were found between girls and boys for MPD (2.38 ± 1.84 and 2.11 ± 1.59, respectively, p = 0.6330), PDC (1.38 ± 0.73 and 1.86 ± 0.95, respectively, p = 0.1034) or CID (0.81 ± 0.52 and 0.90 ± 0.84, respectively, p = 0.7142).

**Table 4.** Comparison of residual spaces measured in millimetres at follow-up on the hemisection and the extraction side.

Variable	Hemisection (N = 40) Mean ± SD	Extraction (N = 40) Mean ± SD	95% CI	P-value
Molar-premolar distance (MPD)	2.04 ± 1.67	2.39 ± 1.86	-0,71, 0.004	0.0525
Premolar-canine distance (PCD)	1.80 ± 1.01	1.55 ± 0.92	0.005, 0.48	0.0455
Canine-incisor distance (CID)	0.88 ± 0.83	0.84 ± 0.77	-0.17, 0.26	0.695
MPD + PCD	3.84 ± 1.97	3.95 ± 2.27	-0.57 ± 0.36	0.6446

A multiple regression analysis was performed to analyse which factors affected the size of the residual space. Premolar angle explained 16% of the variance in PDC (p = 0.0010, *R*^2^ = 0.16) and a positive association was found between the molar angle and CID, explaining 11.5% of the variance in CID (p = 0.0309, *R*^2^ = 0.115). No relationships were found between the residual space (MPD, PDC, and CID) and the patient’s age (p = 0.3997, p = 0.3059 and p = 0.2669, respectively), the root development of either the second molar (p = 0.2257, p = 0.4476 and p = 0.4407, respectively) or the first premolar (p = 0.8178, p = 0.3161 and p = 0.9758, respectively), the vertical position of the second molar (p = 0.6981, p = 0.6916 and p = 0.7025, respectively), or the presence or absence of the third molar (p = 0.2920, p = 0.1555 and p = 0.1961, respectively).

### Secondary outcomes

#### Angulation

There was no statistically significant difference in tipping of the first permanent molar (molar angle) or the first permanent premolar (premolar angle) into the extraction space on the hemisection and extraction side (p = 0.0914 and p = 0.7812, respectively) (Table 5). The vertical relationship of the second molar did not affect the molar angle (p = 0.7180) or the premolar angle (p = 0.4351). The molar angle was significantly larger in boys than in girls (81.16 ± 5.93, 76.68 ± 7.72, respectively, p = 0.0035) while no difference was seen for the premolar angle (112.57 ± 5.12, 112.16 ± 6.10, respectively, p = 0.7461).

**Table 5.** Comparison of the angulation of the first permanent molar and premolar at baseline (T1) and follow-up (T2) between the hemisection and the extraction side.

Variable	T1				T2			
	Hemisection (N = 40) Mean ± SD	Extraction (N = 40) Mean ± SD	95% CI	P-value	Hemisection (N = 40) Mean ± SD	Extraction (N = 40) Mean ± SD	95% CI	P-value
Molar angle (θ)	86.24 ± 5.57	85.55 ± 4.96	-0.82, 2.20	0.3591	82.30 ± 7.14	80.94 ± 6.45	-0.23, 2.95	0.0914
Premolar angle (γ)	102.39 ± 6.35	102.48 ± 7.62	-1.59, 1.41	0.9010	114.07 ± 5.38	114.30 ± 5.63	-1,94, 1,47	0.7812

#### Symptoms and complications

Pain from the remaining mesial root segment following hemisection was reported by 22.5% (n = 9) of the patients. In comparison, 5% (n = 2) of patients experienced post-extraction symptoms after conventional extraction. The difference in pain was statistically significant (p = 0.0176), indicating that hemisection is associated with a higher prevalence of symptoms compared with conventional extraction. No complications were reported during the hemisection or extraction.

## Cost minimisation analysis

Since hemisection and extraction produced identical outcomes, a cost-minimisation analysis was used to identify the most cost-effective option. Descriptive statistics of treatment duration and costs are presented in Table 6. The total number of required visits was one for conventional extraction and 7.25 ± 2.42 for hemisection. The mean chair time and total time parents were absent from work was significantly greater for hemisection than conventional extraction (p < 0.0001 and p < 0.0001, respectively). Thus, the mean indirect cost of hemisection was €169.55, SD = 49.55), significantly higher compared to the €37.72, SD = 3.79) recorded for conventional extraction (p < 0.0001). The mean direct material costs were significantly higher for hemisection than for conventional extraction (p < 0.0001). Similarly, the chair time cost for hemisection (€177.34, SD = 37.89) was significantly greater than for conventional extraction (€61.94, SD = 14.99, p < 0.0001). Consequently, the total direct cost for hemisection was significantly higher, with a mean of €191.84, SD = 39.58), compared with €69.94, SD = 14.99) for conventional extraction (p < 0.0001).

**Table 6.** Descriptive statistics of treatment duration and time (hours) and treatment costs (in €).

	Extraction (n = 40) Mean ± SD	Hemisection (n = 40) Mean ± SD	p-value	95% CI
Chair time (hours)	0.90 ± SD 0.22	2.60 ± SD 0.55	<0.0001	1.5135, 1.8865
Duration of parents’ absence from work (hours)	1.90 ± SD 0.22	9.85 ± SD 2.88	<0.0001	7.0408, 8.8592
Direct cost—material (€)	8.45	14.49 ± SD 2.09	<0.0001	5.3821, 6.6979
Direct cost—chair time (€)	61.49 ± SD 14.99	177.34 ± SD 37.89	<0.0001	103.0235, 128.6765
Direct cost (total in €)	69.94 ± SD 14.99	191.84 ± SD 39.58	<0.0001	108.5008, 135.2992
Indirect cost (€)	37.72 ± SD 3.78	169.55 ± SD 49.55	<0.0001	121.1873, 152.4727
Societal cost (€)	102.66 ± SD 103.74	361.38 ± SD 363.54	<0.0001	139.7166, 377.7234

The total societal cost for hemisection was significantly higher (€361.38, SD = 363.54) than for conventional extraction (€102.66, SD = 103.74; p < 0.0001).

## Harms

More patients reported pain from the remaining mesial root segment after hemisection compared to after conventional extraction.

## Discussion

This study aimed at comparing two techniques in patients with congenitally missing mandibular second premolars, addressing a knowledge gap identified in a systematic review by Naoumova et al. (2017) [10]. The primary findings showed no clinically significant differences in space closure or changes in tooth angulation between the two treatment options. However, hemisection was associated with a higher rate of complications and much higher costs.

The findings of the study provided further insights into factors influencing the size of the residual space when the deciduous molar is removed as a treatment option in cases with agenesis of the lower second premolars. No significant associations were found between residual space and patient age, root developmental stages, vertical position of the second molar, or presence of the third molar. These findings may, however, be attributed to the homogeneity of the study cohort. Most participants were within a narrow age range when diagnosis of agenesis becomes reliable. A broader age range could have offered greater variability to detect age-related effects but that would have involved patients for whom the optimum time frame for an uncomplicated interceptive treatment would have passed. Early diagnosis is important but challenging, sometimes due to late onset of mineralisation of the second premolar buds [1]. Similarly, the uniformity of root development and vertical position may have limited the ability to detect significant relationships, with most participants displaying Nolla stages 5-6 and vertical positions 2-3.

Parallels can be drawn to the extraction of the first permanent molars and the assessment of space closure, as highlighted in a recent meta-analysis by Hamza et al. (2024) [16]. This study demonstrated that space closure in the mandible is generally less successful than in the maxilla. The analysis emphasised that extracting the first permanent molar before the age of ten, when the second permanent molar is at Demirjian stage E (equivalent to Nolla stage 7), and the presence of the third permanent molar significantly improves the likelihood of spontaneous space. Most patients in our study met these optimum conditions concerning age and root development stage, as described in the meta-analysis.

Findings from previous studies by Lindqvist (1980) and Mamopoulou et al. (1996) provide important insights into residual space and tooth angulation outcomes after extraction of the deciduous second molars. Lindqvist reported that spontaneous space closure accounted for about 84% of the extraction site, mainly through mesial migration of the first permanent molar. While the article does not specify if these results apply solely to the mandible, as extractions were performed in both jaws, the findings suggest that after a 4-year follow-up, the mean residual space is 2 mm in the mandible and less than 1mm in the maxilla. Mamopoulou et al. observed a mean residual space of 2.0 mm in the mandible four years after extraction, suggesting significant space reduction. Both studies also evaluated tipping and angulation, reporting a slight mesial tipping of the adjacent teeth in most cases. These findings align with our results, where the mean residual space was 2.0 mm for hemisection and 2.4 mm for conventional extraction, i.e. ~ 82% space closure (presuming that a primary molar is around 11 mm), including minor tipping of the first permanent molar and premolar [6, 7].

There are no randomised controlled trials to date comparing hemisection and conventional extraction as interceptive treatments for mandibular second premolar agenesis. While the existing literature on hemisection suggests potential advantages, such as preserving alveolar bone and promoting parallel tooth movement, these findings primarily derive from descriptive studies and case control studies. Valencia et al. (2004) investigated hemisection as a method to promote mesial movement of the mandibular molars while reducing anterior tipping. Their study compared outcomes for patients treated with hemisection and those who underwent extraction of the primary molars. Although hemisection appeared to result in improved outcomes, particularly in younger patients aged 8–9 years, this age range is too early for a definitive diagnosis of premolar agenesis. Furthermore, the criteria used to classify results as good, average, or poor were unclear, and the small sample size, lack of a power analysis, and the imprecise follow-up period limit the reliability and applicability of the findings. Similarly, Northway et al. (2004) demonstrated the previously described advantages of hemisection. However, a limitation of this study is that the reported treatment outcomes may not be entirely attributable to the hemisection technique alone, as some form of orthodontic intervention was used to facilitate final space closure. Once the first permanent molar had drifted mesially to the point where further movement was restricted, the study incorporated various anchorage protection appliances—such as an activator, Jasper Jumper, Herbst appliance, protraction face mask, or Hickham chin cup—to maintain anterior tooth position. In addition, fully banded or bonded appliances were used for final space closure. This makes it difficult to isolate the effects of hemisection itself from the influence of orthodontic mechanics, potentially limiting the reliability of the conclusions drawn. Although promising, their findings are limited by the absence of a direct comparison with conventional extraction and the lack of long-term follow-up to assess the stability of treatment outcomes. When comparing these observations with our findings, it becomes evident that while hemisection may have theoretical advantages, it does not yield superior clinical outcomes regarding space closure or tooth angulation compared with conventional extraction [8, 9].

As hemisection and conventional extraction resulted in equivalent primary clinical outcomes, a cost minimisation analysis was performed. Economic evaluations of orthodontic treatments offer valuable insights for effective planning and management of orthodontic services [17]. A comparison of societal costs revealed that hemisection of the mandibular primary second molars was 3.5 times more expensive than conventional extraction. Hemisection necessitates two appointments to extract the same tooth, whereas conventional extraction requires only one. Furthermore, prior to extraction of the mesial root, several appointments were required to assess whether the space was less than 2 mm. Consequently, hemisection involves more visits, increased utilisation of clinical resources, and higher material and chair time costs. Additionally, a larger number of appointments results in more absence from work for parents, contributing to higher indirect costs due to loss of production hours and wages. This study further identified significantly more complaints from patients associated with hemisection.

## Strengths and Limitations

This study has several strengths, including its prospective design, split-mouth methodology and extended follow-up period. Additionally, a blinded operator ensured impartial outcome measurements, while a comprehensive evaluation of multiple factors, including space closure, angulation, complications and cost was conducted. However, some limitations must be acknowledged. The absence of baseline models precluded analysis of changes in arch form and arch parameters. Furthermore, patient-reported outcomes were derived solely from record data, limiting insight into subjective experiences. The assessment of indirect costs was not based on precise measurements of productive hours lost or income lost by the parents of the participants. Distributing surveys to collect detailed data from parents would provide a more accurate representation of the differences in indirect costs between the two intervention groups. Nevertheless, given the clear findings that hemisection resulted in greater complications without yielding superior outcomes in space closure or angulation, additional patient-reported data would unlikely alter the conclusions. Hemisection was also found to be much more costly than conventional extraction.

## Generalisability

The findings of this study are generalisable to a similar cohort of patients with bilateral agenesis of mandibular second premolars, matched by age and root development stage of the permanent second molar. The split-mouth design strengthens the internal validity, reducing inter-individual variability. Economic evaluations are inherently influenced by the economic framework, cost of dental materials, and healthcare worker salary structures in a specific country. Therefore, these findings may not be directly generalisable to countries with different economic systems

## Clinical Implications and Future Research

The findings suggest that conventional extraction should remain the preferred interceptive treatment in cases of mandibular second premolar agenesis. Hemisection offers no advantage and presents greater risks and costs. Post-operative symptoms were reported more frequently following hemisection, particularly regarding the remaining mesial root segment.

Hemisection also requires an additional appointment with administration of local anaesthesia and removal of the remaining half of the tooth, potentially leading to increased dental anxiety or fear, especially in younger patients. This additional injection increases the treatment burden and highlights the need for careful consideration of the child’s psychological readiness and ability to tolerate extended procedures. Based on our results, we can expect a mean residual space of 2.0 mm along with a mean four-degree tilting of the permanent first molar following interceptive extraction of the primary second molar prior to the eruption of the second permanent molar. Additionally, a space opening mesially of the first permanent premolar (1.8 mm) can be excepted. The interceptive extraction and spontaneous space closure facilitates potential subsequent orthodontic treatments, avoiding the need for extensive interventions such as implants or retention of the primary molar, which may have a questionable long-term prognosis. Although Bjerklin et al. (2008) reported a generally favourable prognosis for retained primary molars, several disadvantages have been highlighted, including their limited lifespan and potential complications [10, 18].

Future research should aim to assess the histological condition of the remaining hemisectioned root, potential variations in alveolar bone volume on the two sides and the impact of interceptive extraction on dentoskeletal and soft tissue changes in the long term. These aspects are currently being investigated, and the findings are expected to be published in the near future.

## Conclusions

- The result of this study shows that there are no clinically significant differences in space closure or changes in tooth angulation between conventional extraction and hemisection.
- Hemisection is associated with more symptoms and complaints from patients and leads to higher direct and indirect costs than conventional extraction.

## Data Availability

All data generated or analysed during this study are included in this article. Further enquiries can be directed to the corresponding author.
